# Conformational flexibility in carbapenem hydrolysis drives substrate specificity of the class D carbapenemase OXA-24/40

**DOI:** 10.1016/j.jbc.2022.102127

**Published:** 2022-06-14

**Authors:** Joshua M. Mitchell, Cynthia M. June, Vincent L. Baggett, Beth C. Lowe, James F. Ruble, Robert A. Bonomo, David A. Leonard, Rachel A. Powers

**Affiliations:** 1 Department of Chemistry, Grand Valley State University, Allendale, Michigan, USA; 2 Research Service, Louis Stokes Cleveland Department of Veterans Affairs Medical Center, Cleveland, Ohio, USA; 3 Departments of Medicine, Biochemistry, Molecular Biology and Microbiology, Pharmacology, and Proteomics and Bioinformatics, Case Western Reserve University School of Medicine, Cleveland, Ohio, USA; 4 CWRU-Cleveland VAMC Center for Antimicrobial Resistance and Epidemiology (Case VA CARES), Cleveland, Ohio, USA

**Keywords:** carbapenem, lactamase, class D, OXA-24/40, X-ray structure, CHDL, carbapenem-hydrolyzing class D β-lactamase, PDB, Protein Data Bank

## Abstract

The evolution of multidrug resistance in *Acinetobacter* spp. increases the risk of our best antibiotics losing their efficacy. From a clinical perspective, the carbapenem-hydrolyzing class D β-lactamase subfamily present in *Acinetobacter* spp. is particularly concerning because of its ability to confer resistance to carbapenems. The kinetic profiles of class D β-lactamases exhibit variability in carbapenem hydrolysis, suggesting functional differences. To better understand the structure–function relationship between the carbapenem-hydrolyzing class D β-lactamase OXA-24/40 found in *Acinetobacter baumannii* and carbapenem substrates, we analyzed steady-state kinetics with the carbapenem antibiotics meropenem and ertapenem and determined the structures of complexes of OXA-24/40 bound to imipenem, meropenem, doripenem, and ertapenem, as well as the expanded-spectrum cephalosporin cefotaxime, using X-ray crystallography. We show that OXA-24/40 exhibits a preference for ertapenem compared with meropenem, imipenem, and doripenem, with an increase in catalytic efficiency of up to fourfold. We suggest that superposition of the nine OXA-24/40 complexes will better inform future inhibitor design efforts by providing insight into the complicated and varying ways in which carbapenems are selected and bound by class D β-lactamases.

Carbapenems are an important antibiotic therapy for difficult-to-treat infections. Carbapenems are essential for a wide variety of medical uses because of their ability to effectively target a broad spectrum of Gram-negative bacteria and a more narrow spectrum of Gram-positive bacteria (1). However, the rise of carbapenem resistance is a recent trend that has resulted in an international crisis (2), especially with the recent isolation of colistin-resistant strains of *Acinetobacter*. To date, novel plasmid-mediated colistin resistance vectors (*mcr*) are identified, for example, *mcr-1* and *mcr-2* (3, 4). These genes are found throughout the world, from the initial identification in China, to Belgium, and the United States, and will likely continue to be discovered. Widespread resistance to traditional broad-spectrum antibiotics could indicate entry into a “postantibiotic” era.

A major focus within the medical community is on pathogens that exhibit multidrug resistance, such as *Acinetobacter* spp (5). These bacteria are notoriously difficult to treat in clinical settings because of a suite of resistance mechanisms that may include efflux pumps, porin channel mutations, β-lactamases, and/or altered penicillin-binding proteins. The most prevalent resistance mechanism to β-lactam antibiotics is expression of β-lactamase enzymes, and all four classes of β-lactamases are identified in *Acinetobacter* (6, 7). Classes A, C, and D are serine β-lactamases that use serine as the nucleophile in a classical two-step acylation/deacylation mechanism. Class D enzymes are distinct in that they utilize a carboxylated lysine as the general base in both steps, activating the catalytic serine residue in acylation and the water molecule for deacylation.

Of particular concern are the carbapenem-hydrolyzing class D β-lactamases (CHDLs) found in *Acinetobacter*, such as those of the OXA-23, OXA-24/40, and OXA-51 subfamilies, that effectively hydrolyze these very potent antibiotics (8). Their clinical significance is quite apparent in the literature, which highlights their presence globally (2, 9). A novel carbapenem inactivation mechanism has been reported whereby CHDLs overcome inhibition *via* formation of a lactone product with carbapenems containing a 1β-methyl group. This was first observed with OXA-23 and OXA-48 (10), and more recently, with OXA-24/40 (11). Traditional β-lactam–based β-lactamase inhibitors are ineffective against these enzymes, and inhibition by the newer boronic acid non-β-lactam–based inhibitor vaborbactam is minimal at best (12). The rise of CHDLs has significantly limited the available treatments for *Acinetobacter* spp. infections.

The CHDL OXA-24/40 is prominently distributed in *Acinetobacter baumannii* and is characterized by the presence of a hydrophobic bridge comprised of Tyr112 and Met223 that covers the active site (13, 14). The bridge distinguishes this enzyme (and OXA-23) from other class D carbapenemase subfamilies (like OXA-48) (15) and has been implicated in playing a role in carbapenem binding and/or carbapenemase activity (13, 14, 16). In general, OXA-24/40 displays tight binding affinities for carbapenems, with *K*_S_ values ranging from 24 nM (for doripenem) up to 752 nM (for imipenem) (17). In contrast, expanded-spectrum cephalosporins are considered poor substrates for class D enzymes (18).

To gain insight into the CHDL OXA-24/40 from *A. baumannii*, we kinetically characterized the enzyme’s ability to hydrolyze the carbapenems meropenem and ertapenem and compared them with doripenem and imipenem, as well as the cephalosporin cefotaxime. We then determined the X-ray crystal structures of OXA-24/40 in complexes with imipenem, meropenem, ertapenem, doripenem, and cefotaxime (Fig. 1). This structure–function analysis reveals ways in which OXA-24/40 interacts with carbapenems that help to explain its preference for carbapenem substrates compared with expanded-spectrum cephalosporins. Comparisons between these nine structures provide a comprehensive view of the active site of OXA-24/40 and its interactions with carbapenem ligands that can inform efforts to design β-lactams and β-lactamase inhibitors.

**Figure 1:**
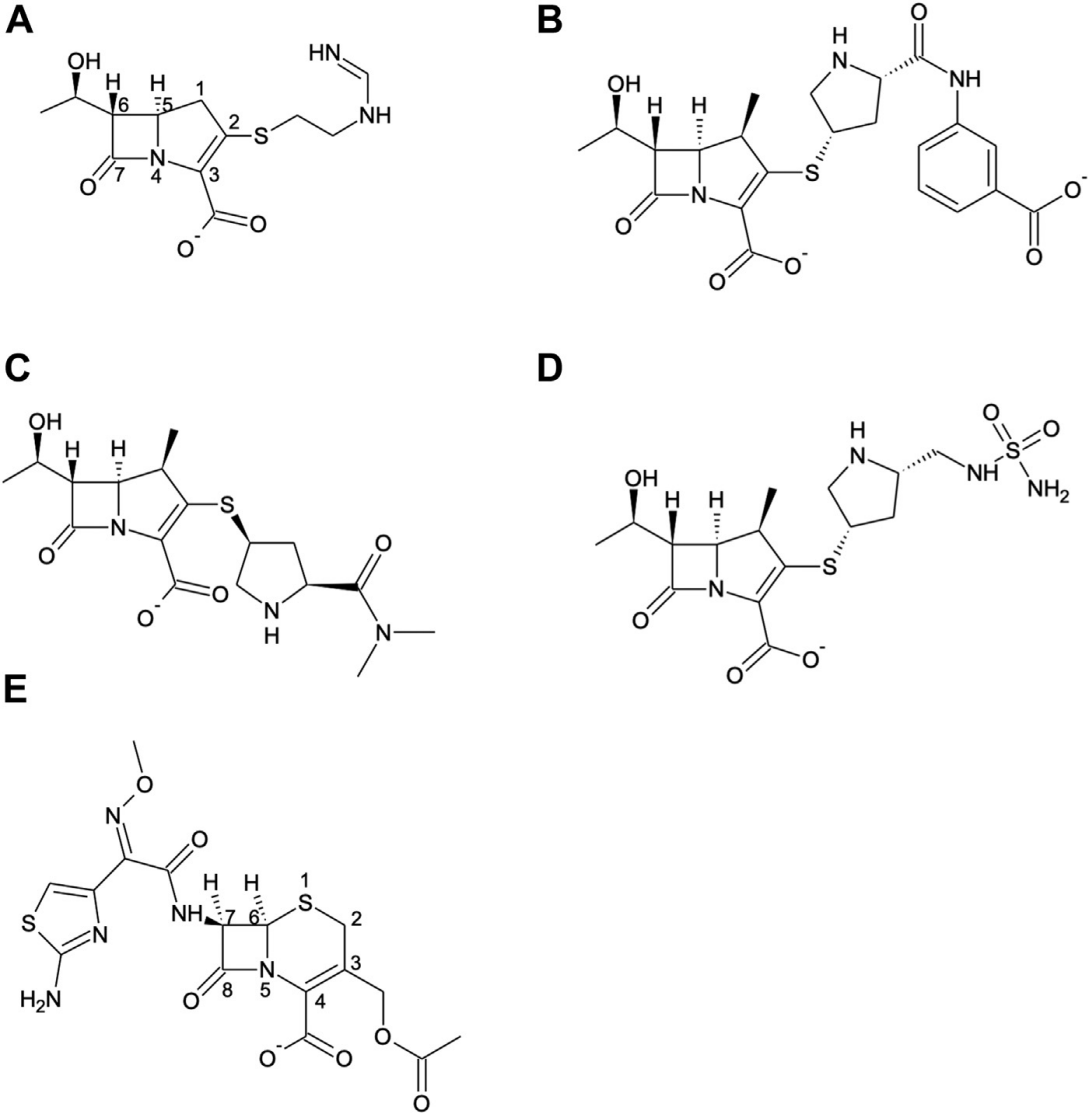
**Structures of β-lactamase ligands.***A*, imipenem. *B*, meropenem. *C*, ertapenem. *D*, doripenem. *E*, expanded-spectrum cephalosporin cefotaxime.

## Results

### Kinetics

Steady-state kinetic characterization of *wt*OXA-24/40 was performed with meropenem and ertapenem (Table 1). In general, carbapenems bind with high affinity to OXA-24/40 (17, 19), and *K*_*m*_ values cannot be accurately calculated through the Michaelis−Menten−Henri equation. Therefore, we measured how well various carbapenem substrates compete with a reporter substrate ampicillin using a technique that is often used to determine reversible competitive inhibitor affinity constants (*K*_s_). Carbapenems can form acyl complexes and are thus not true reversible inhibitors, but this method has been widely used to estimate proxy values for relative affinity of slow substrates (20, 21, 22, 23). In order to acknowledge that the *K* value is not a true affinity constant, we refer to it as *K*_s_∗ (Equation 1). The hydrolysis profile of OXA-24/40 showed that ertapenem and meropenem have similar profiles to doripenem (*K*_S_∗ = 24 nM and *k*_cat_ = 0.074 s^−1^). Both bind with nanomolar affinities (*K*_S_∗ values of 6.9 and 18 nM for ertapenem and meropenem, respectively) and have relatively low turnover rates (*k*_cat_ values of 0.093 s^−1^ and 0.131 s^−1^ for ertapenem and meropenem, respectively). To investigate this further, crystallographic studies were pursued to analyze structural features that could elucidate the reasons for the observed activity.

**Table 1:** OXA-24/40 steady-state kinetic parameters

β-lactam antibiotic	*K*_*M*_ or *K*_S_∗ (μM)	*k*_cat_ (s^−1^)	*k*_cat_/*K*_*M*_ (μM^−1^·s^−1^)
Ampicillin^a^	180 ± 20	480 ± 20	2.6 ± 0.3
Cefotaxime^a^	750 ± 70	0.38 ± 0.01	0.00050 ± 0.00005
Imipenem^a^	0.752 ± 0.10^b^	2.1 ± 0.04	2.8 ± 0.4
Doripenem^a^	0.024 ± 0.003^b^	0.074 ± 0.001	3.1 ± 0.4
Meropenem	0.018 ± 0.003^b^	0.131 ± 0.005	7.3 ± 1.1
Ertapenem	0.0069 ± 0.001^b^	0.093 ± 0.006	13.5 ± 2.1

a Previously reported in the study by Kaitany *et al.* (17).

b *K*_S_∗ values were determined by competition kinetics with ampicillin as a reporter substrate.

### Crystallography

A total of seven X-ray crystal structures of OXA-24/40 bound to imipenem, meropenem, and ertapenem were determined to resolutions ranging from 2.58 to 1.53 Å resolution (Table S1). Two deacylation-deficient variants of OXA-24/40 (K84D and V130D) were employed to capture acyl–enzyme complexes with the substrates (14). A structure of *wt*OXA-24/40 in complex with doripenem was also determined to 1.90 Å resolution. To elucidate the basis for the weak cephalosporinase activity noted in previous studies (17), the structure of OXA-24/40 K84D bound to cefotaxime was also determined to 1.62 Å resolution. All complexes crystallized in the same space group (P4_1_2_1_2) with similar cell parameters and one molecule in the asymmetric unit (Table S1). The quality of the models was evaluated using the Protein Data Bank (PDB) evaluation server (24).

In all the structures, initial *F*_0_–*F*_c_ electron density maps (contoured at 3σ) indicated the presence of the β-lactams bound in the active site. In general, the cores of the carbapenems were observed more clearly in the initial electron density than the side chains at the C2 position, which suggested that the C2 groups may be flexible or exist in multiple conformations. Polder omit maps were calculated to confirm the conformations of the ligands in the final models (Fig. S1).

**Figure 2:**
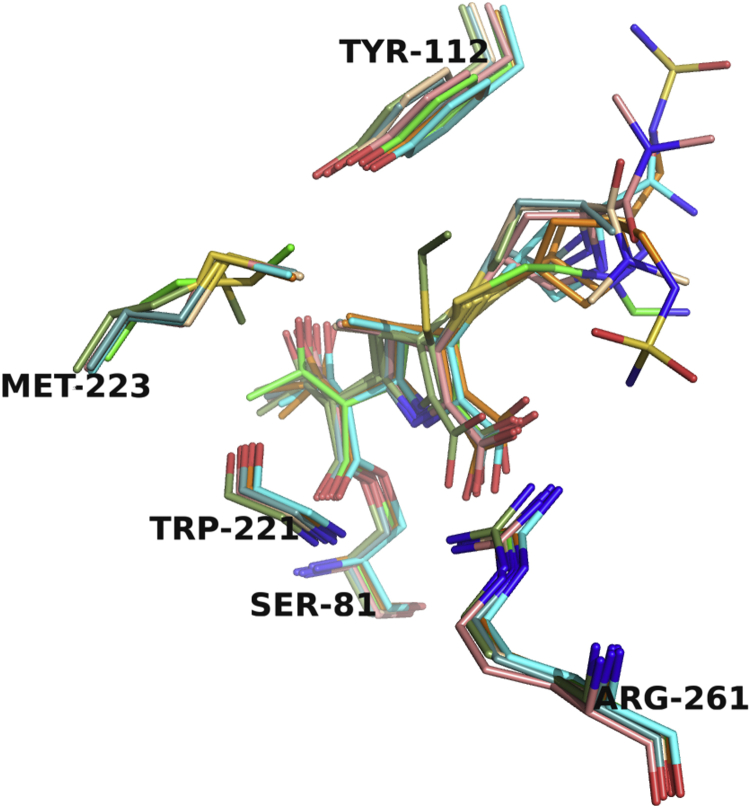
**Superposition of OXA-24/40 complexes with carbapenems.** Imipenem (K84D, *green*; V130D, *smudge*), ertapenem (wt, *cyan*; V130D, *light teal*), meropenem (K84D, *salmon*; V130D, *wheat*), and doripenem (wt, *orange*). The oxyanion hole is indicated with the main-chain atoms of Ser81 and Trp221 (side-chain atoms are removed for clarity). The C3/4 carboxylate recognition residue is Arg261. The intact hydrophobic bridge unique to OXA-24/40 is composed of Tyr112 and Met223. Oxygen atoms are colored *red*, nitrogens *blue*, sulfurs *yellow*, and carbon atoms for each of the complexes are colored as indicated above. This figure and all subsequent ones were generated with PyMOL (42).

Overall, several interactions are maintained in all the complexes (Fig. 2). The acyl-carbonyl oxygen of the ligand is bound in the oxyanion hole and makes its expected hydrogen bonds with the main chain -NH groups of Ser81 and Trp221. In addition, the C3/4 carboxylate group forms interactions with Arg261. A defining feature of OXA-24/40 is the presence of a hydrophobic bridge, comprised of Tyr112 and Met223, that extends over the active site (13). Previous research suggests that this bridge may explain the high affinity binding of carbapenems since it forms favorable van der Waals interactions with the C2 side chains of these molecules (13, 14). Others have proposed a role for the bridge in influencing the tautomerization state of the carbapenem (14). In all the structures presented here, the bridge is not disrupted upon ligand binding and remains intact.

**Supplemental Figure S2:**
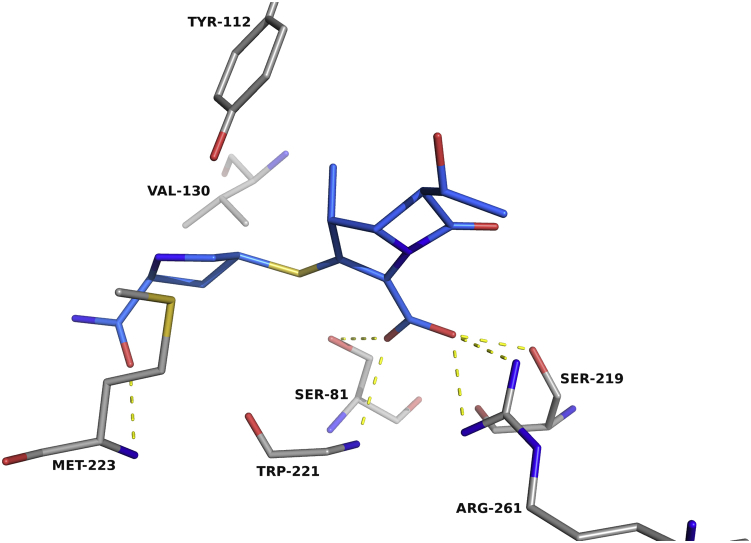
**Unproductive binding mode of ertapenem bound to OXA-24/40.** Hydrogen bonding and ionic interactions are indicated with *yellow dashed lines*. Carbon atoms of ertapenem are colored *marine blue*, and carbon atoms of OXA-24/40 are colored *white*. The intact β-lactam ring and hydroxyethyl group of ertapenem are *circled*.

Acyl–enzyme complexes for all the carbapenems were captured with both of the OXA-24/40 deacylation–deficient variants (K84D and V130D) and are described in more detail later. One exception was the structure of OXA-24/40 K84D with the carbapenem ertapenem, which resulted in an intact β-lactam captured in an apparently unproductive binding mode (Figs. S1*E* and S2). Additional attempts at capturing an acyl–enzyme complex with this variant were unsuccessful. Therefore, the structure of *wt*OXA-24/40 with ertapenem was obtained.

#### OXA-24/40 complexes with imipenem

A predominant conformation for imipenem was observed in the K84D complex, although there is some evidence of multiple conformations of the C2 side chain, suggesting flexibility of this portion of the molecule (Fig. S1, *A* and *B*). The entire imipenem was modeled in the predominant conformation at an occupancy of 0.70. For the V130D complex, imipenem appears to sample two conformations, which are modeled at occupancies of 0.7:0.3. Electron density for the three distal atoms of the C2 side chain was not observed, and these atoms are not present in the final model. The main difference between each of these complexes is the placement of the C2 side chain, which regardless of conformation, makes few interactions within the active site.

**Figure 3:**
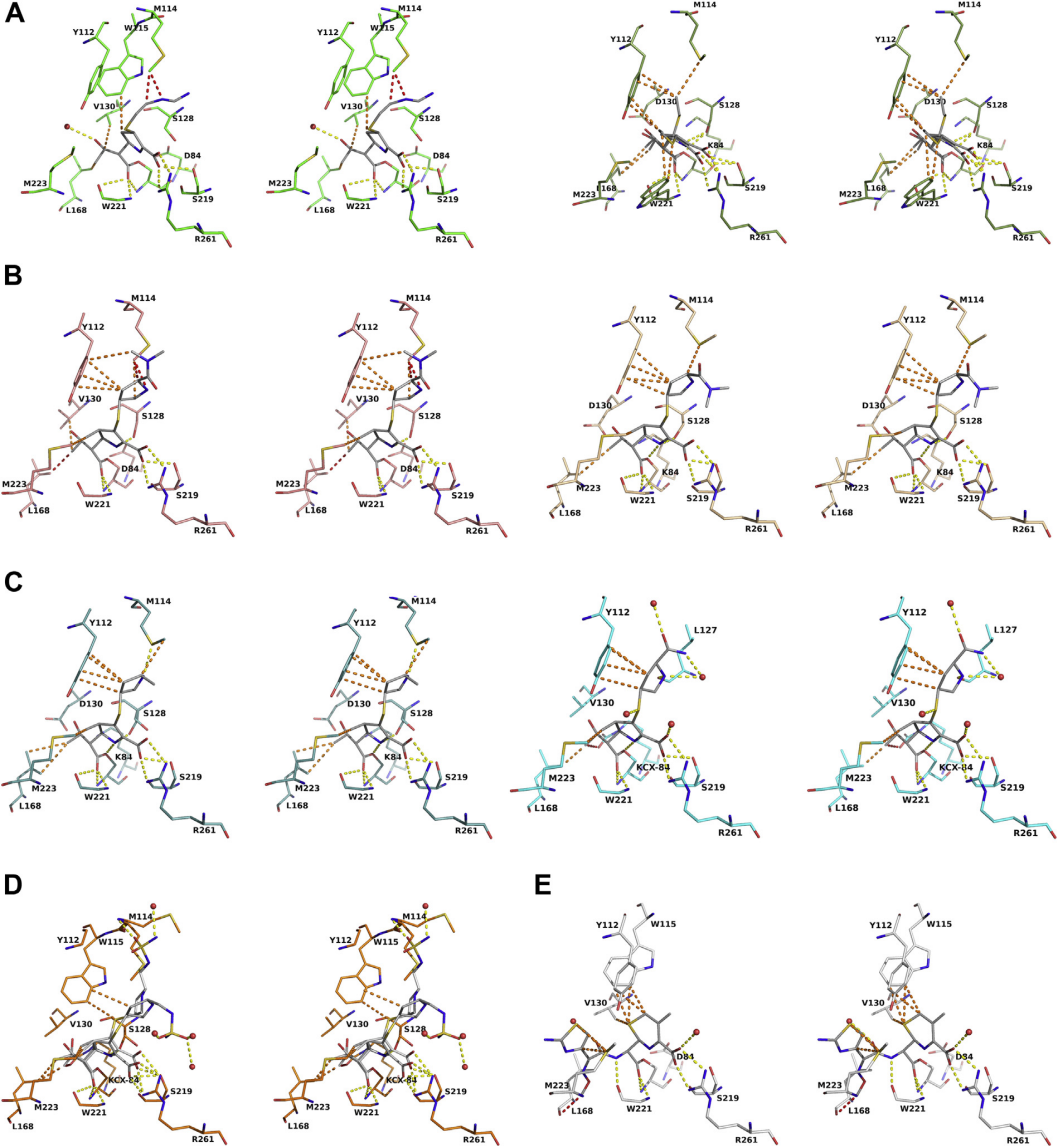
**Walleye stereoviews of active sites of OXA-24/40 with bound β-lactam ligands.***A*, imipenem (K84D, *top*; V130D, *bottom*). *B*, meropenem (K84D, *top*; V130D, *bottom*). *C*, ertapenem (wt, *top*; V130D, *bottom*). *D*, doripenem (wt). *E*, cefotaxime (K84D). Carbon atoms for the ligands are colored *gray* to distinguish them from the residues in the active site, which are colored as indicated for Figure 2. Water molecules are drawn as *red spheres*. Hydrogen bonds and ionic interactions are indicated with *dashed yellow lines* for distances between 2.5 and 3.2 Å; van der Waals interactions are shown as *orange dashed lines* for distances between 3.3 and 4.0 Å; *clashes* are shown as *red dashed lines*.

**Supplemental Figure S3:**
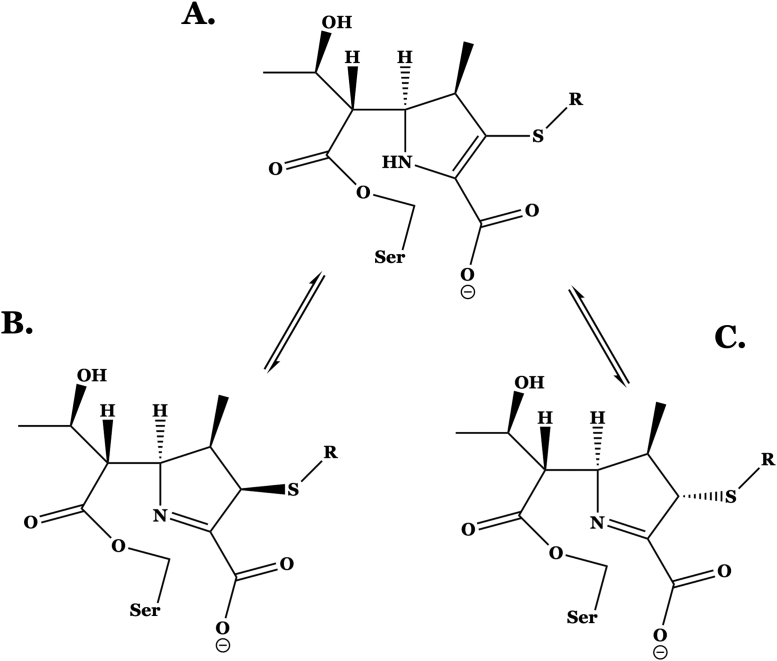
**Structures of a generic acylated carbapenem shown in its different tautomeric conformations.***A*, Δ^2^ tautomer. *B*, Δ^1^R tautomer. *C*, Δ^1^S tautomer.

Carbapenems are unique in that they undergo a significant structural rearrangement after acylation that results in their observed biphasic kinetics (25, 26, 27). The pyrroline double bond between carbons C2–C3 can tautomerize to C3–N4, resulting in two isoforms (Δ^2^ and Δ^1^, respectively), with the Δ^2^ tautomer deacylated more rapidly than the Δ^1^ tautomer (Fig. S3) (25, 27). In both the imipenem complexes, the pyrroline ring adopts the Δ^1^ tautomer. A single Δ^1^R tautomer is observed in the K84D complex, but in the V130D complex, the pyrroline ring appears to sample both the Δ^1^R and Δ^1^S tautomers (Fig. 3*A*).

**Figure 4:**
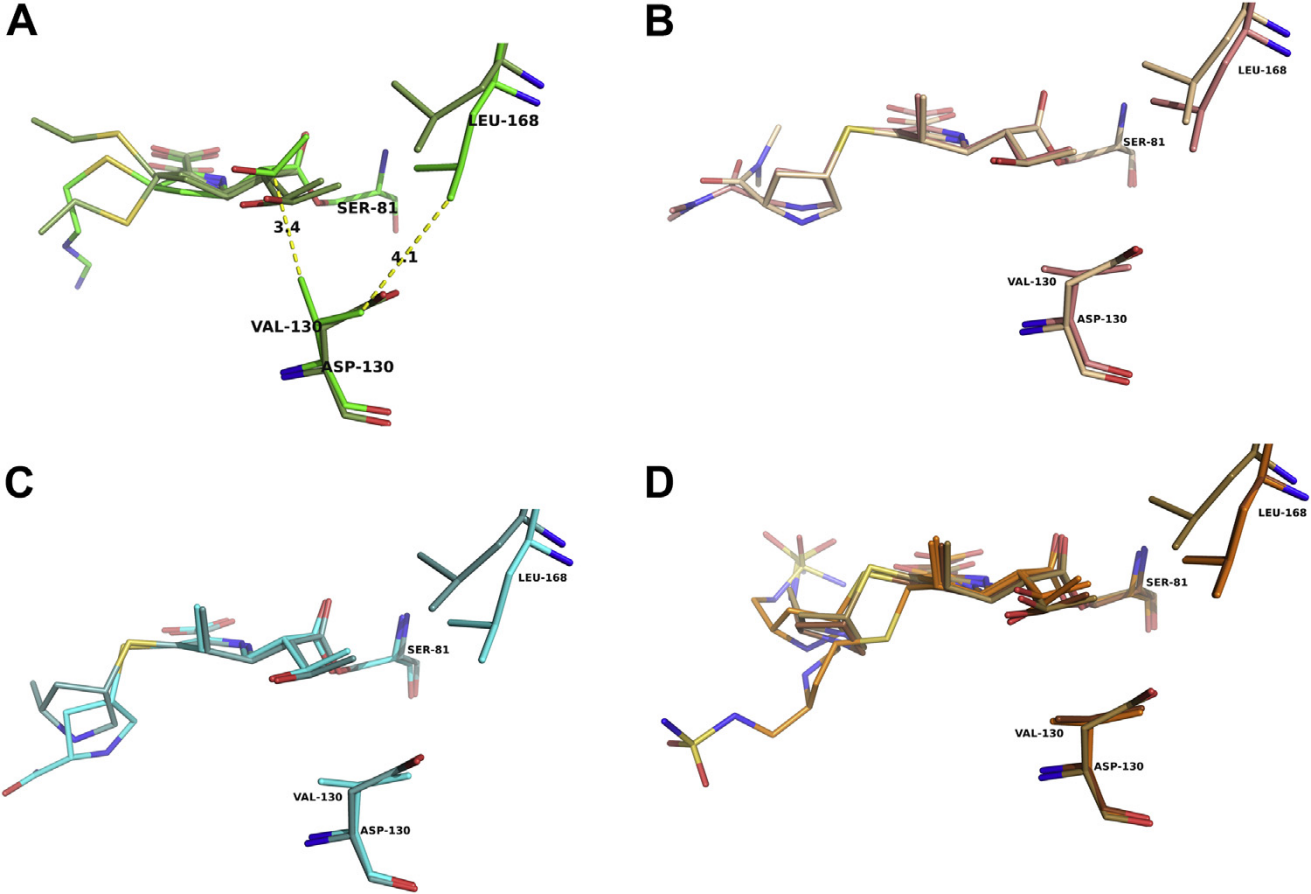
**Structural differences observed in the carbapenem complexes of the OXA-24/40 V130D variant.***A*, imipenem complexes with V130D and K84D variants. *B*, meropenem complexes with V130D and K84D variants. *C*, ertapenem complexes with V130D and WT. *D*, doripenem complexes with V130D and WT. The V130D/doripenem structure (Protein Data Bank [PDB] ID: 3PAG) is shown with carbon atoms colored *brown*. The conformation of the hydroxyethyl group in the K84D/imipenem complex (*top*; in *green*) is tipped back away from the “closed” conformation of Val130 to avoid a steric clash. Also shown is the conformation of Leu168 that is consistently observed in the V130D complexes to avoid a clash with the larger aspartate substitution as this position (*upperright* of each image).

**Figure 5:**
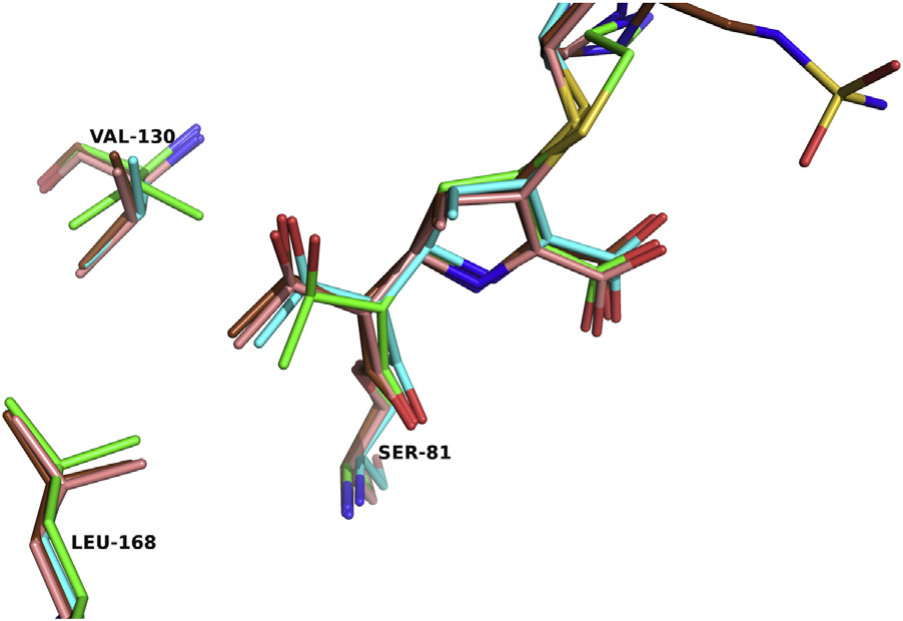
**Superposition of OXA-24/40 K84D complexes with carbapenems.** Val130 is found in a “closed” conformation in the K84D–imipenem complex (*green*) but is “open” in the other carbapenem complexes. The K84D–doripenem complex (Protein Data Bank [PDB] ID: 3PAE) is included in this comparison with carbon atoms colored *brown*.

The hydroxyethyl group of imipenem adopts a similar rotamer in both complexes, although in the K84D complex, this group has noticeably tipped away from Val130 (Fig. 4). Val130 has been implicated in regulating entry of the deacylating water to the active site by rotating between an “open” state that allows entry of the water to the point of deacylation and a “closed” state in which access is blocked (20). In the K84D complex, Val130 adopts a “closed” conformation that positions its Cγ2 atom toward the imipenem hydroxyethyl group (∼3.4 Å) and atom Cγ1 toward Leu168 (∼4.1 Å) (Fig. 5). This “closed” state results in the observed shift of the hydroxyethyl group of imipenem away from Val130 to avoid a steric clash.

#### OXA-24/40 complexes with meropenem

In both complexes, the pyrroline ring of meropenem is observed in the Δ^2^ tautomer, and the hydroxyethyl group is in the same conformation. In contrast to the imipenem complex, Val130 is rotated into an “open” conformation, where Val130Cγ2 is now oriented toward Leu168 (∼4.0 Å) and away from the hydroxyethyl group of meropenem (Figs. 3*B* and 4). The C2 side chains of meropenem are not making any interactions within the active sites and exhibit high B-factors, again suggesting that the C2 side chains of the carbapenems are flexible.

#### OXA-24/40 complexes with ertapenem

Acyl–enzyme complexes of ertapenem with both *wt*OXA-24/40 and the V130D variant were determined. Ertapenem binds in a similar manner as meropenem, with the pyrroline ring adopting the Δ^2^ tautomer and the hydroxyethyl group in similar conformations (Fig. 3*C*). Weak electron density prevented modeling of the entire C2 side chains of ertapenem, again suggesting flexibility of this part of the molecule and making few, if any, interactions with the enzyme. In the final model of V130D, atoms beyond the pyrrolidine ring of ertapenem are not included, and in the wt complex, the atoms of the terminal benzoic acid group are not present. The *wt*OXA-24/40 complex with ertapenem presents us with what is presumed to be a functional active site. Indeed, Lys84, the general base in the mechanism, is carboxylated (Kcx84), which is required for efficient deacylation (28), and Val130 is observed in an “open” conformation (Fig. 3*C*, *top* and Fig. 4). However, a candidate for a deacylating water molecule is not observed near Kcx84. In the unproductive K84D–ertapenem complex, Val130 is in its “closed” conformation.

#### OXA-24/40 complex with doripenem

We previously determined the structures of OXA-24/40 K84D and V130D in complexes with doripenem (PDB ID: 3PAE and 3PAG, respectively) (14). Here, we were able to capture an acyl–enzyme complex of doripenem bound to *wt*OXA-24/40. In both the variants, the pyrroline ring is in the Δ^2^ tautomer. In the wt structure, the electron density suggests doripenem samples both the Δ^2^ and Δ^1^S tautomers, with the doripenem modeled in two conformations (occupancies of 0.5; Fig. 3*D*). In the Δ^2^ tautomer, the C2 side chain follows a similar trajectory to that previously observed in the variants, but the trajectory is different in the Δ^1^S tautomer. Finally, Val130 is in an “open” state, but as with the *wt*OXA24-40/ertapenem complex, no deacylating water molecule is observed in proximity to Kcx84 (Figs. 3*D* and 4).

#### OXA-24/40 complex with cefotaxime

The complex of OXA-24/40 K84D with the expanded-spectrum cephalosporin cefotaxime was determined to provide insight on the weak cephalosporinase activity of OXA-24/40. Cephalosporins contain substituents at both the C3 and C7 positions. In this complex, the thiazolidine ring of cefotaxime binds in a similar location as the pyrroline ring of the carbapenems, and the group at the C3 position has been eliminated to form a stable acyl–enzyme intermediate (Fig. 3*E*). On the other side of the molecule, the C7 substituent of cefotaxime is much larger and in the opposite stereochemistry when compared with the hydroxyethyl group of the carbapenems, which causes this group to take a different trajectory in the active site. The amide carbonyl oxygen in the C7 substituent is oriented toward Val130; in fact, this oxygen is ∼3.5 Å from Val130Cγ2. Val130 adopts the “closed” state in this complex.

## Discussion

Analysis of the nine complexes of carbapenems bound to the CHDL OXA-24/40 provides a consensus view of how this enzyme recognizes its substrates and suggests possible structural reasons for observed differences in activity (Fig. 2). Functionally, the carbapenem that is noticeably distinct from the others is imipenem, which is the smallest as compared with the other carbapenems (Fig. 1). The flexible C2 side chain made relatively few interactions with active-site residues, consistent with its weaker binding affinity. However, what accounts for the ability of OXA-24/40 to turnover imipenem approximately 15-fold to 30-fold faster than the other carbapenems tested? A distinguishing feature among these carbapenems is the substituent at the C1 position. The newer carbapenems (ertapenem, meropenem, and doripenem) contain a β-methyl group at this position, which was introduced to make the acyl–enzyme intermediate more resistant to hydrolysis. Imipenem lacks the 1β-methyl group and is hydrolyzed more rapidly by OXA-24/40.

The structures revealed another interesting feature regarding the uniqueness of imipenem when compared with the rest of the carbapenems: the tautomeric state of the pyrroline ring. The majority of the complexes of OXA-24/40 with ertapenem, meropenem, and doripenem show that the Δ^2^ tautomer is predominant, although doripenem adopts both the Δ^2^ and Δ^1^S tautomers in the *wt*OXA-24/40 complex. However, in both imipenem complexes, the Δ^1^R tautomer is observed, with a mixture of Δ^1^R and Δ^1^S for the V130D complex. Previous work in OXA-23 showed that for carbapenems containing large C2 side chains (*i.e.*, doripenem and meropenem), the bridge directed them into either the Δ^2^ tautomer or the Δ^1^S tautomer and prohibited formation of the Δ^1^R (16). Carbapenems with smaller C2 groups, like imipenem, would not be impacted as much and might therefore be able to sample the Δ^1^R tautomer. With the bridge intact in these complexes, the apparent flexibility of imipenem allows OXA-24/40 to efficiently hydrolyze it.

A degree of active-site flexibility on the part of the enzyme also appears to be necessary for carbapenemase activity. The active site of *wt*OXA-24/40 has been suggested to adopt distinct conformations that influence whether hydrolysis or β-lactonization will be the predominant mechanism. Lactone formation appears to depend on the presence of Arg261 (11). Entry of the deacylating water molecule is regulated by a hydrophobic clamp comprised of residues Val130 and Leu168 in OXA-24/40. In previous studies of the CHDL OXA-23, carbapenem binding was shown to induce water channel opening *via* movement of Leu168, whereas computational studies suggested that in OXA-24/40, rotation of Val130 opens the water channel (16, 29). Indeed, in our complexes with meropenem, ertapenem, and doripenem, Val130 is found in an “open” state, presumably induced by a clash with the hydroxyethyl groups upon acylation. Comparison with the imipenem complex revealed that imipenem is again unique among the carbapenems with the conformation of Val130 in the “closed” state, yet imipenem is turned over approximately 20-fold to 30-fold faster than doripenem and meropenem (Fig. 4 and Table 1). To reconcile these findings, a structural comparison reveals a unique binding mode for imipenem, with its hydroxyethyl group tipped away from Val130, avoiding a clash with this residue (closest distance of 3.4 Å). This conformation is not adopted by any of the larger carbapenems. The smaller and more flexible imipenem may allow for easier access of the deacylating water in the acyl–enzyme complex where no clash is present. Active-site flexibility is also observed on the part of the enzyme with Val130 adopting alternate conformations in the same structure (*i.e.*, both “open” and “closed”) in several OXA-24/40 complexes with boronic acid inhibitors (PDB IDs: 5TG4 and 5TG7) (30). Its ability to sample both conformations may also help facilitate efficient hydrolysis.

One particular carbapenem complex proved to be unique. In the K84D–ertapenem structure, ertapenem is bound as an intact β-lactam, yet in an unproductive binding mode that would not allow for acylation. This binding mode was reproducible only with the K84D variant and only with ertapenem. Perhaps this static structure is an indication that carbapenem hydrolysis by class D enzymes involves the sampling of multiple nonproductive binding modes before a productive conformation is identified for catalysis. Alternatively, this binding mode might be suggestive of ertapenem acting as both an inhibitor, when in its unproductive mode, and a substrate, when in its catalytically competent conformation.

In contrast to the carbapenems, why are expanded-spectrum cephalosporins extremely poor substrates for OXA-24/40? The catalytic efficiencies of cefotaxime and ceftriaxone are very low (0.00050 and 0.00030 μM^−1^ s^−1^, respectively), deriving mostly from the weak binding affinities (*K*_*m*_ = 750 and 114 μM), and OXA-24/40 showed no measurable binding or turnover of ceftazidime (17). Inspection of the OXA-24/40 complex with cefotaxime provides some insight. We superposed this complex with that of OXA-160 V130D in complex with ceftazidime (PDB ID: 4X56). OXA-160 is essentially equivalent to OXA-24/40, except for a single substitution of Pro227Ser. In these complexes, the C7 substituents of the cephalosporins are rotated ∼180° from each other. The smaller oxyimino group of cefotaxime is accommodated in the active site such that its aminothiazole ring makes van der Waals interactions with bridge residue Met223. If ceftazidime were to adopt the same conformation, its bulkier oxyiminodimethylcarboxylate group would clash with Met223, which likely explains the better binding affinity of cefotaxime for OXA-24/40.

**Supplemental Figure S4:**
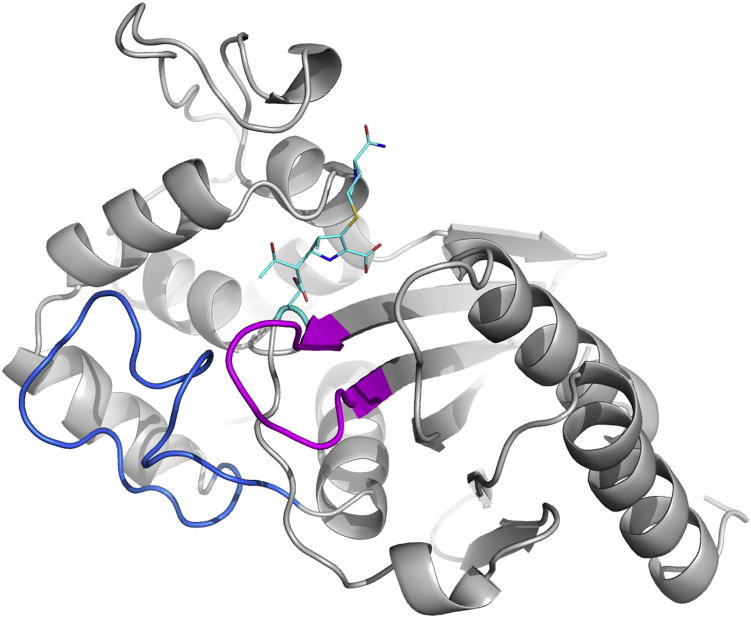
**Overview of the entire OXA-24/40 structure.** The active site is indicated by an acylated ertapenem bound to the catalytic Ser81, with carbons of both colored *cyan*. Two loops surround the active site. The Ω loop (residues 154–174) is colored *marine blue*, and the β5–β6 loop (residues 221–230) is colored *magenta*.

Further inspection of the OXA-24/40 complex with cefotaxime revealed that changes in the Ω loop structure of the enzyme are not present, but binding of cefotaxime results in a disruption of the β5–β6 loop (Fig. S4). The β5–β6 loop, which contains the bridge residue Met223, has been implicated in the acquisition of carbapenemase activity (31) as well as expanded-spectrum cephalosporinase activity (19). In fact, OXA-160, with its Pro227Ser substitution, becomes a better cephalosporinase as compared with OXA-24/40 (19). The position of Met223 and the hydrophobic bridge itself remain unchanged, but the electron density for residues 224 to 225 is not well defined, suggesting that this region follows a different path than in the rest of the OXA-24/40 complexes.

CHDLs are a significant threat to the continued efficacy of some of our most potent β-lactam antibiotics. Analysis of the crystal structures reported here point to a concerning ability of OXA-24/40 to retain hydrolysis of these drugs regardless of modifications to them. OXA-24/40 has achieved active-site plasticity that complements this variability and flexibility. Although carbapenem antibiotics exhibit altered functionality, the overall enzyme efficiency remains consistent. In-depth inquiry to elucidate the mechanism by which carbapenem-hydrolyzing β-lactamases preserve their utility against carbapenem drugs is a threat that the scientific and medical community cannot afford to ignore. Our structures highlight the versatility of these enzymes and their ability to adapt to antibiotic engineering. The preservation of the catalytic efficiency of OXA-24/40 in the face of structural alterations of carbapenems shows a concerning evolution of β-lactamases toward further resistance to our most potent antibiotics and the possible bleak future of a postantibiotic world. Fortunately, these structures supply us with a wealth of information regarding the active site of OXA-24/40 and its binding capabilities to recognize antibiotics, which can aid in the design of high-affinity β-lactamase inhibitors.

## Experimental procedures

### Protein expression and purification

wt OXA-24/40 as well as the K84D and V130D variants were expressed and purified as previously described (14). Purified protein samples (>95% by Coomassie staining) for kinetics were snap frozen in liquid nitrogen and stored at −80 °C. Protein for crystallization was concentrated to 6 to 10 mg/ml using an Amicon Ultra centrifugal filtration device (10 kDa molecular weight cutoff; Millipore) and used within 48 h. The concentration of all protein samples was determined by measuring the absorbance at 280 nm and using an extinction coefficient calculated by the method of Gill and von Hippel (32).

### Steady-state kinetic analysis

Kinetic parameters were determined on a Carey60 UV–Vis Spectrophotometer by the addition of aliquots of purified *wt*OXA-24/40 to various concentrations of β-lactam substrate in 50 mM NaH_2_PO_4_, 25 mM NaHCO_3_, pH 7.4 at 25 °C. Changes in absorbance as a function of time were converted to velocity (micromolar/second) using the following Δε values (M^−1^ cm^−1^): meropenem, −6500 (λ = 300 nm) and ertapenem, −10,940 (λ = 295 nm). Initial rates from at least three trials were averaged and plotted as a function of substrate concentration. For meropenem and ertapenem, *k*_cat_ values were determined from nonlinear regression of the data to the Michaelis−Menten−Henri equation using the Microsoft Excel SDAS module. The *K*_*m*_ value was too low to be measured accurately by this method, so *K*_S_ values were estimated by using meropenem and ertapenem as competitive inhibitors with ampicillin as a reporter substrate. The concentration of OXA-24/40 used in the competition assays with ertapenem and meropenem was 5 nM, and the concentration of ampicillin used as the reporter substrate was 300 μM. The enzyme was added to a cuvette containing the carbapenem (concentrations ranging from 0 to 2 μM) and ampicillin. Carbapenems were not preincubated with the enzyme. In this case, *K*_S_ values were calculated from the concentration of substrate that generated 50% of uninhibited velocity (IC_50_) using the Cheng−Prusoff equation (33). Because these carbapenems are slow substrates and not reversible inhibitors, the value derived is not a true *K*_S_, and we refer to it as *K*_S_∗.

(1) $$K_{s}^{*}=\frac{{\text{IC}}_{50}}{1+\;\frac{\left[S\right]}{K_{m}}}$$

### Crystallization

Crystals of OXA-24/40 were grown by hanging drop vapor diffusion using well buffer composed of either 0.1 M Tris (pH 8.5), 2.0 M ammonium sulfate, or 0.1 M Hepes sodium (pH 7.5), 2% v/v polyethylene glycol 400, and 2.0 M ammonium sulfate (K84D/imipenem). OXA-24/40 crystallizes equally well in both conditions, and crystals grown in both conditions result in the same space group and cell parameters. Purified protein (6–10 mg/ml) was combined with well buffer in various ratios (1:1–19:1) in a total drop volume of 10 μl, and crystals were formed within 24 h at room temperature. Preformed crystals of OXA-24/40 (K84D, V130D, or wt) were soaked in 10 to 25 mM meropenem, 3 to 25 mM imipenem, 10 to 25 mM ertapenem, 10 to 25 mM doripenem, or 10 mM cefotaxime in well buffer supplemented with 25 mM bicarbonate and 5% sucrose as a cryoprotectant. Soak times ranged from 5 to 15 min after which crystals were immediately flash cooled in liquid nitrogen.

### Structure determination

Diffraction data were measured at the LS-CAT beamline of the Advanced Photon Source at 100 K. Specific details of each data collection experiment are included in the individual PDB submissions. Reflections were indexed, integrated, and scaled using either HKL2000 or autoPROC (34). All the structures were determined by molecular replacement with Phaser (https://www.phaser.cimr.cam.ac.uk/index.php/Phaser_Crystallographic_Software) (35) using the OXA-24/40 K84D structure (PDB ID: 3PAE with all ligand and water molecules removed) as the initial phasing model, except for the K84D–imipenem complex where PDB of 2JC7 was used as the starting model. Refinement and electron density map calculations were carried out with REFMAC5 (https://www.ccp4.ac.uk/html/refmac5/credits.html) (36) in the CCP4 program suite (37). Manual rebuilding of the model was accomplished with Coot (https://www2.mrc-lmb.cam.ac.uk/personal/pemsley/coot/docs/coot-faq.html#Citing) (38). Polder omit maps were calculated with Phenix (39) by omitting the ligand and using a 3.0 Å solvent exclusion radius (40, 41).

## Data availability

The atomic coordinates and structure factors are deposited with the PDB with IDs 7RP8 (K84D/imipenem), 7RP9 (V130D/imipenem), 7RPA (K84D/meropenem), 7RPB (V130D/meropenem), 7RPC (K84D/ertapenem), 7RPD (V130D/ertapenem), 7RPE (wt/ertapenem), 7RPF (wt/doripenem), and 7RPG (K84D/cefotaxime). Authors will release these data upon article publication.

## Supporting information

This article contains supporting information. Crystallographic statistics for the nine OXA-24/40 complexes are provided in Table S1. Polder omit maps for each of the complexes are shown as stereoviews in Fig. S1. Interactions observed in the unproductive complex of K84D OXA-24/40 with ertapenem are shown in Fig. S2. Chemical structures of the different carbapenem tautomers are provided in Fig. S3. An overview of the entire OXA-24/40 enzyme with the Ω and β5–β6 loops indicated is provided in Fig. S4.

## Conflict of interest

The authors declare that they have no conflicts of interest with the contents of this article.

## Supporting information

Supplemental Table S1

Supplemental Figure S1A–H
