# Supplementary material for: Conformational flexibility in carbapenem hydrolysis drives substrate specificity of the class D carbapenemase OXA-24/40
Source: J Biol Chem. 2022 Jun 14;298(7):102127. doi: 10.1016/j.jbc.2022.102127 (PMC9293634; doi:10.1016/j.jbc.2022.102127)

**Supplementary Figure 1.** Walleye stereoviews of the Polder omit maps for OXA-24/40 complexes. Maps were calculated with Phenix on the final models.

1. K84D/imipenem (contoured at 5.0 σ).

**
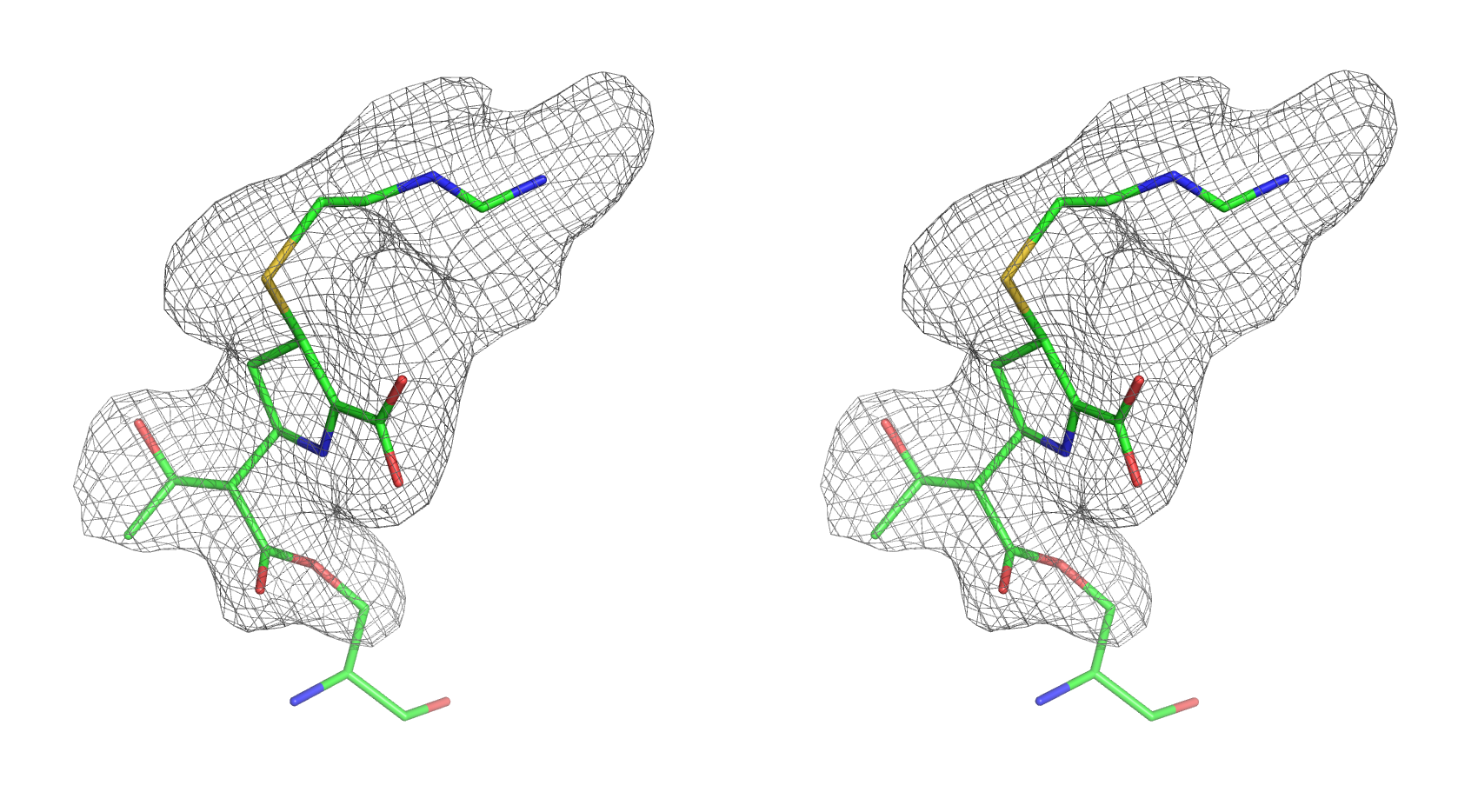
**

1. V130D/imipenem (contoured at 4.5 σ).


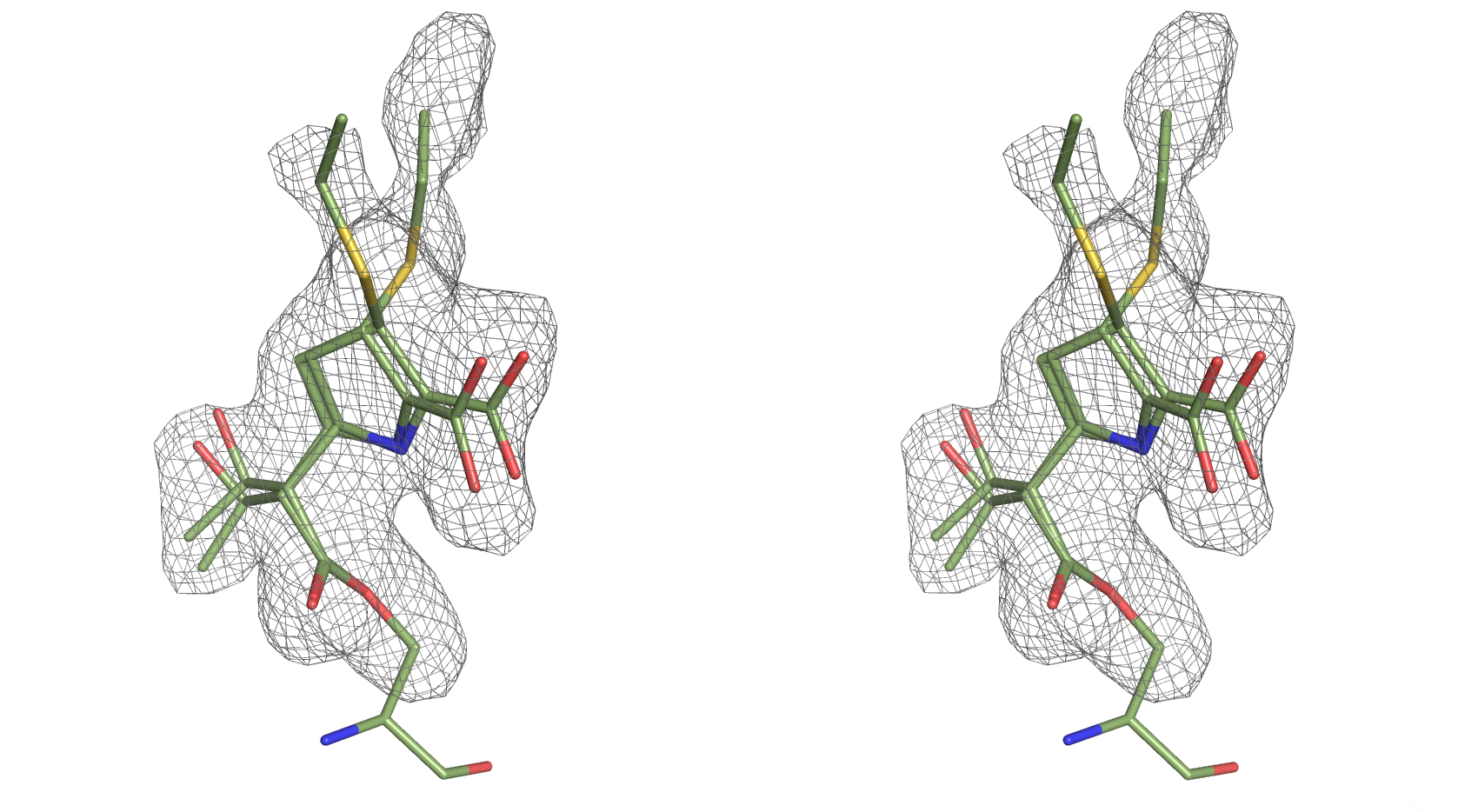


1. K84D/meropenem (contoured at 5.0 σ).


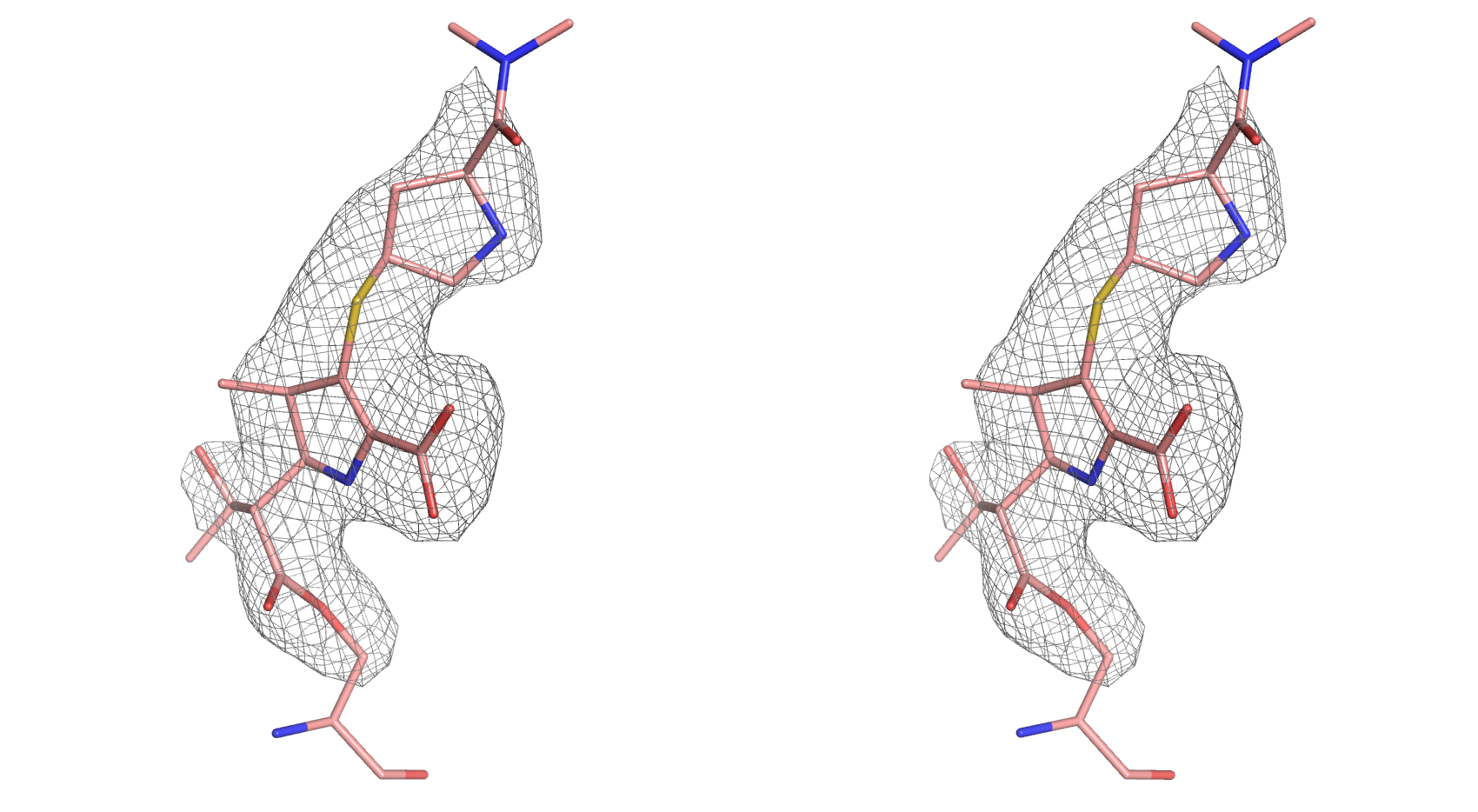


1. V130D/meropenem (contoured at 5.0 σ).


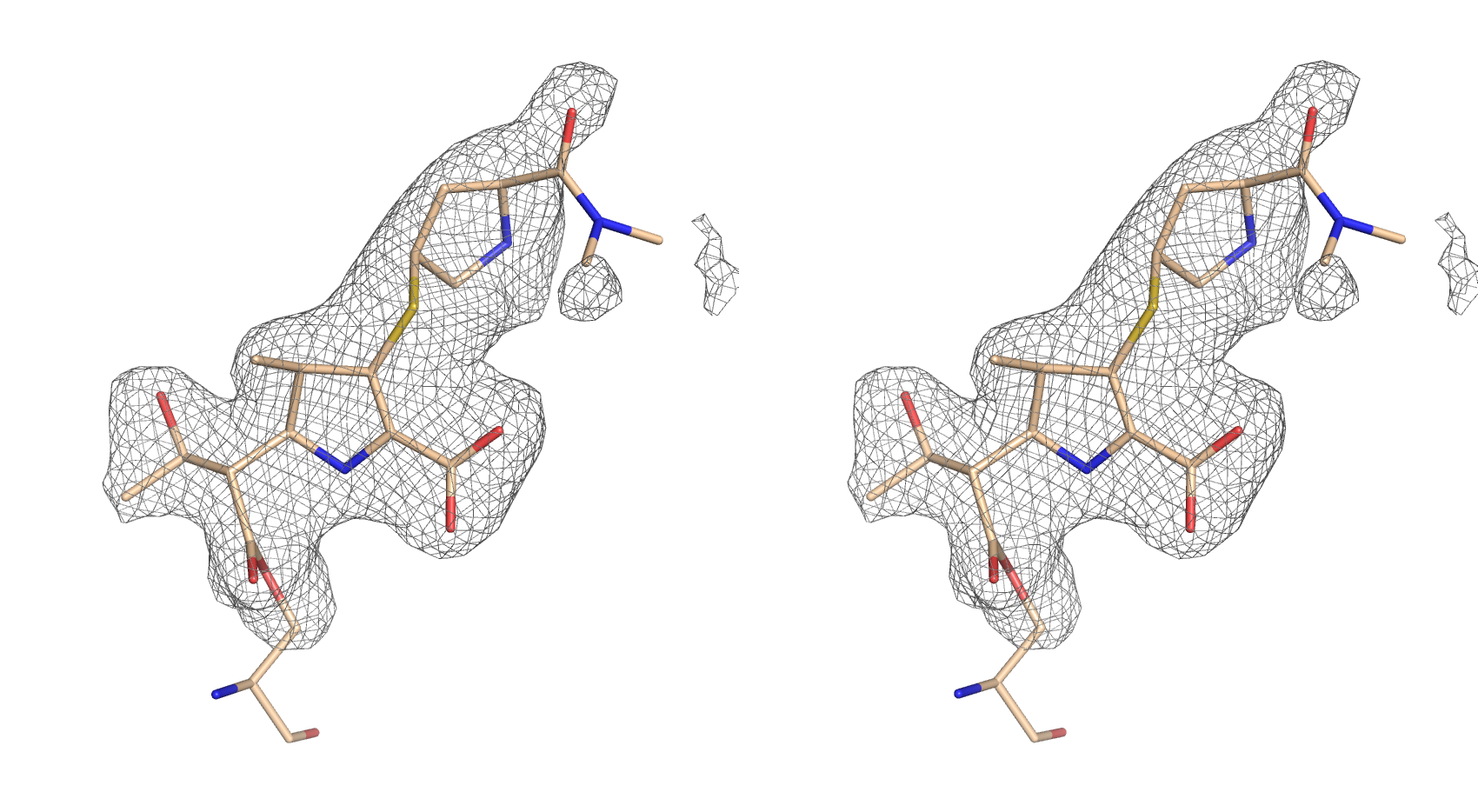


1. K84D/ertapenem (contoured at 4.5 σ).


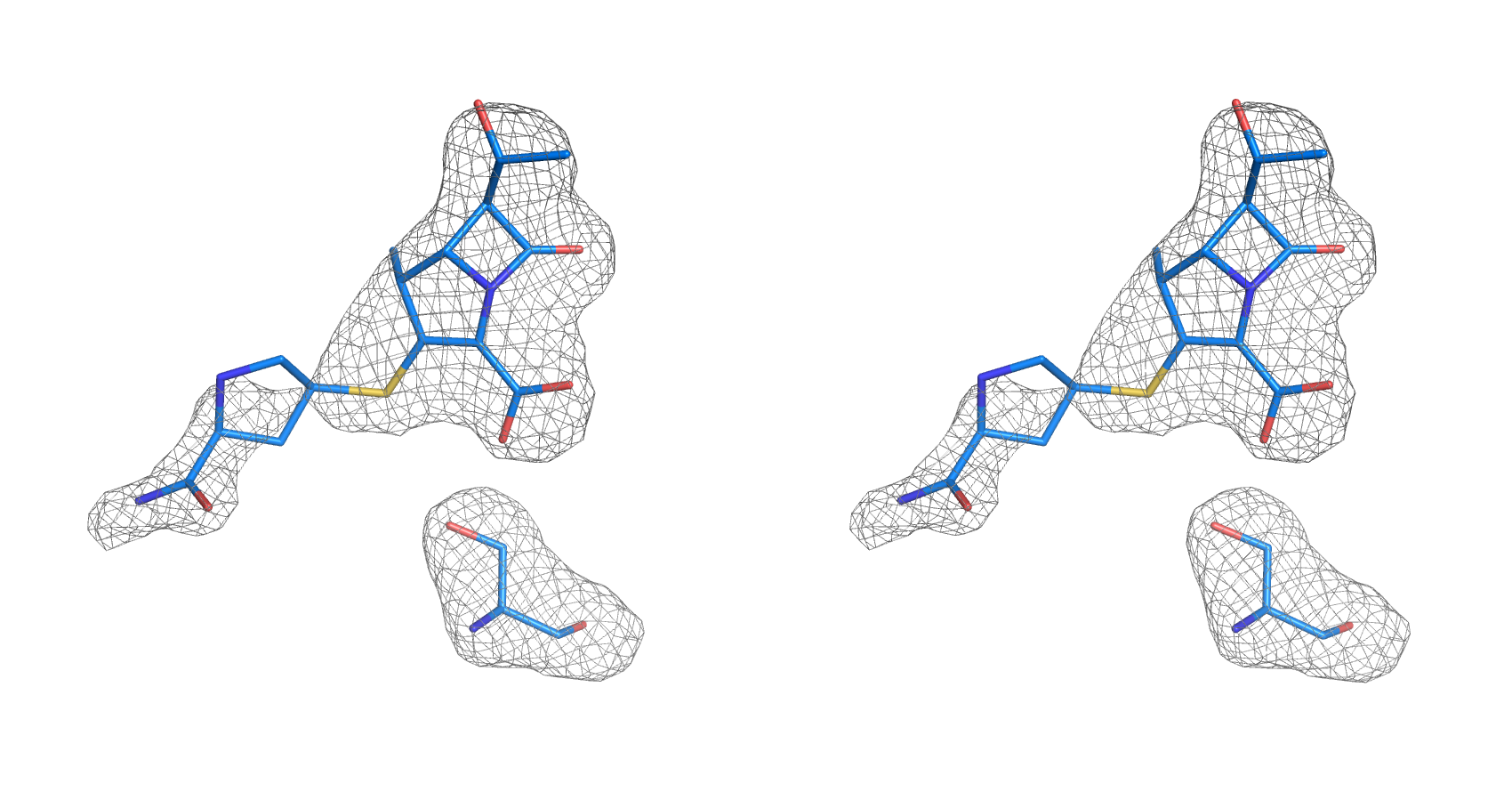


1. V130D/ertapenem (contoured at 5.0 σ).


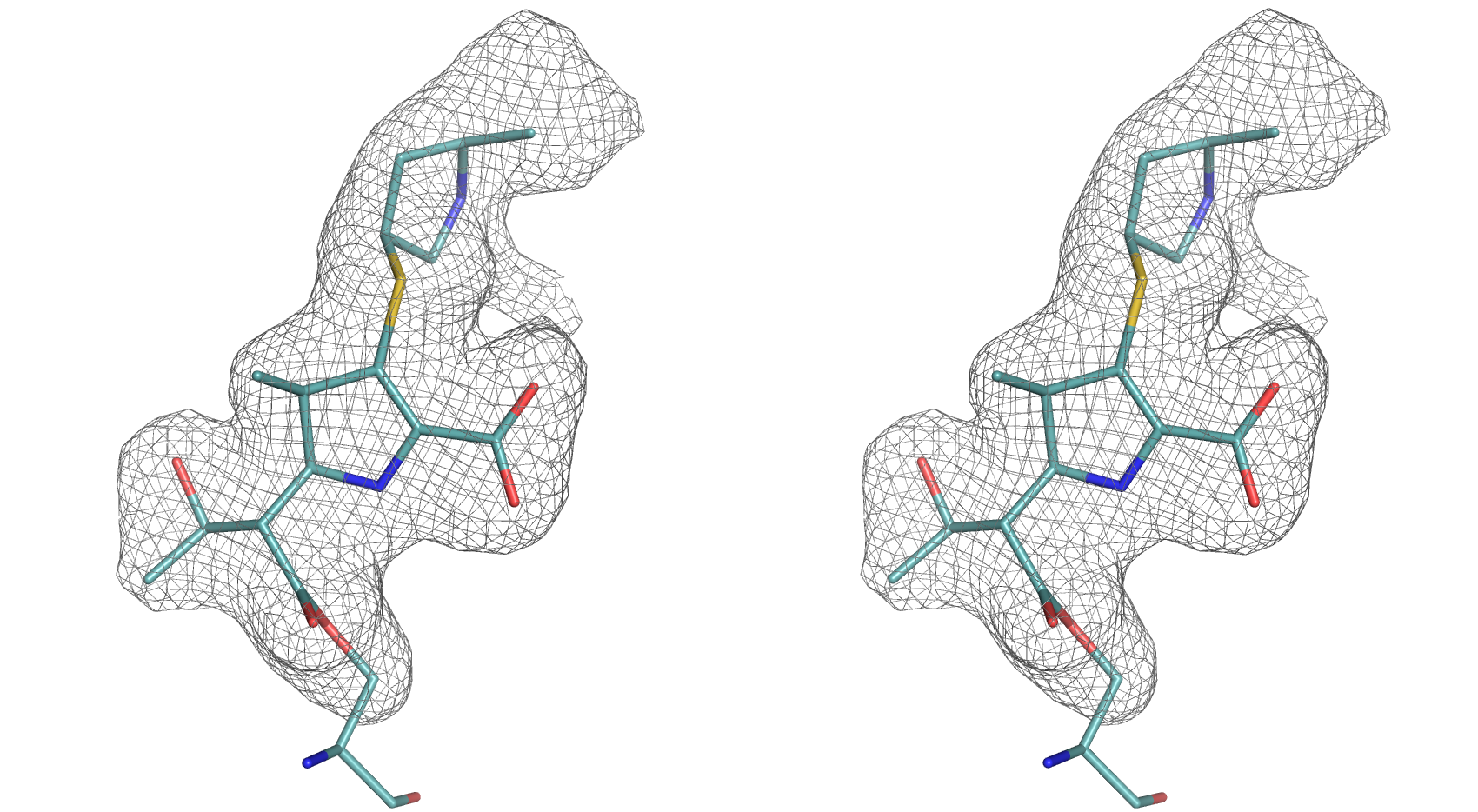


1. WT/ertapenem (contoured at 5.0 σ).


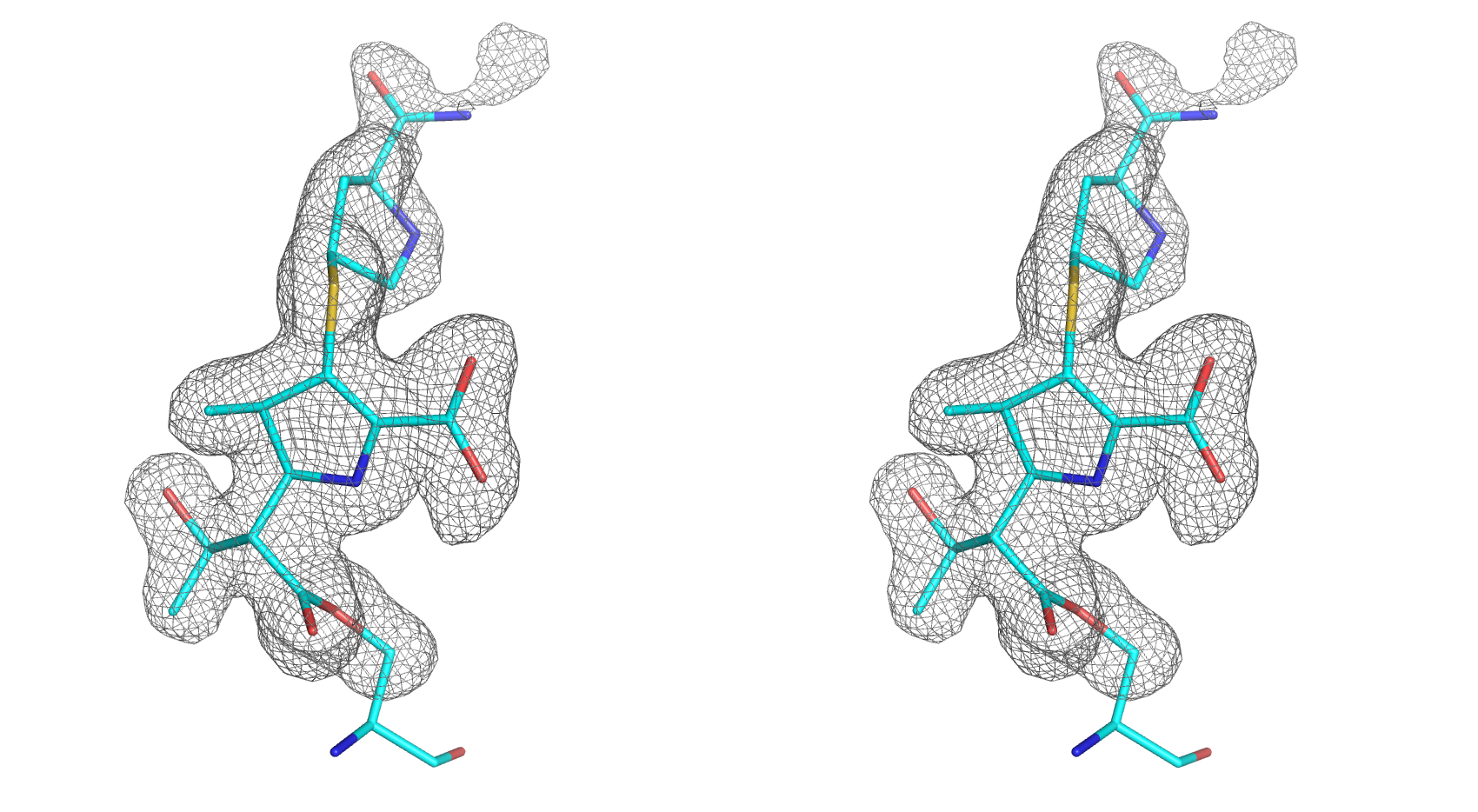


1. WT/doripenem (contoured at 4.5 σ).


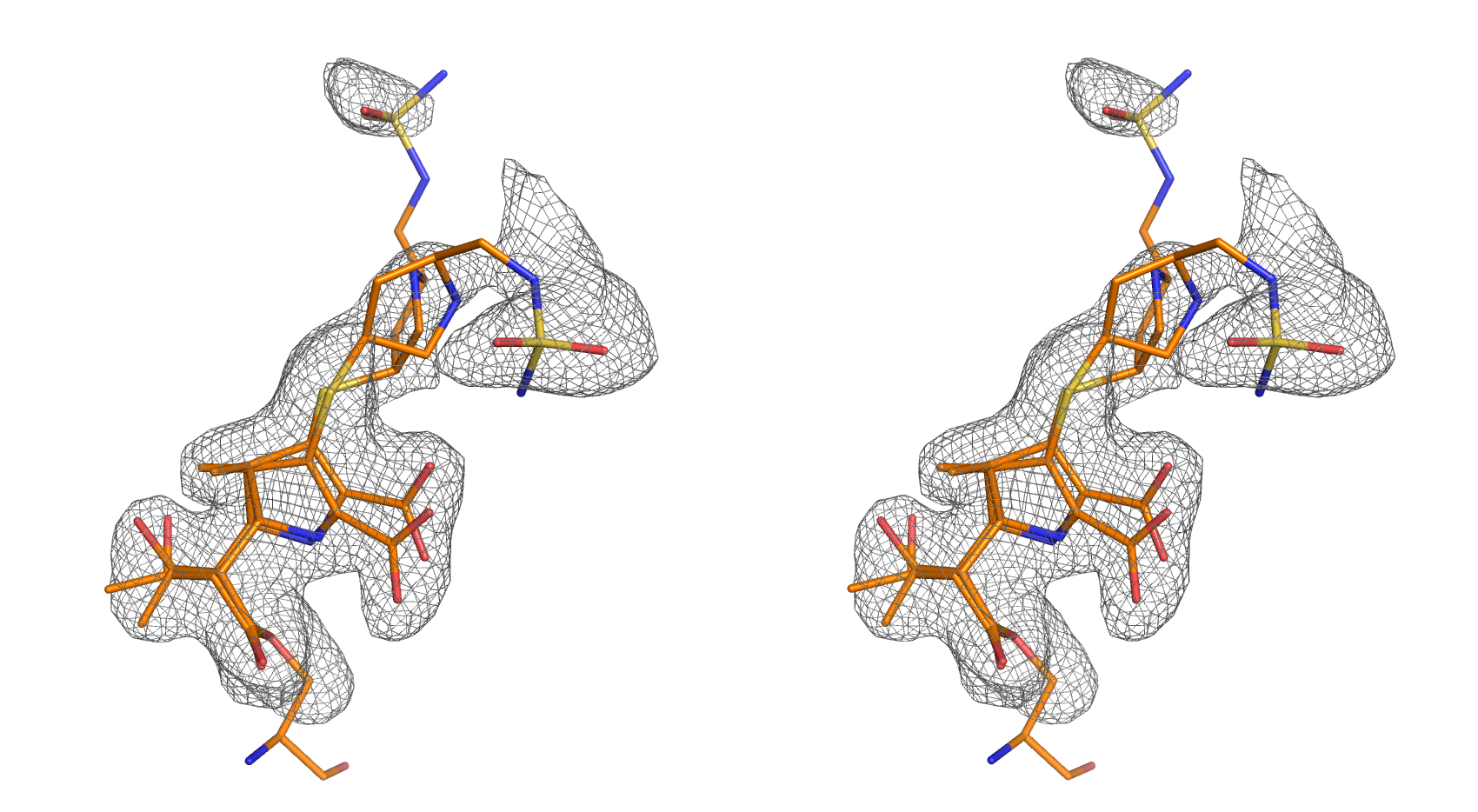


1. K84D/cefotaxime (contoured at 5.0 σ).


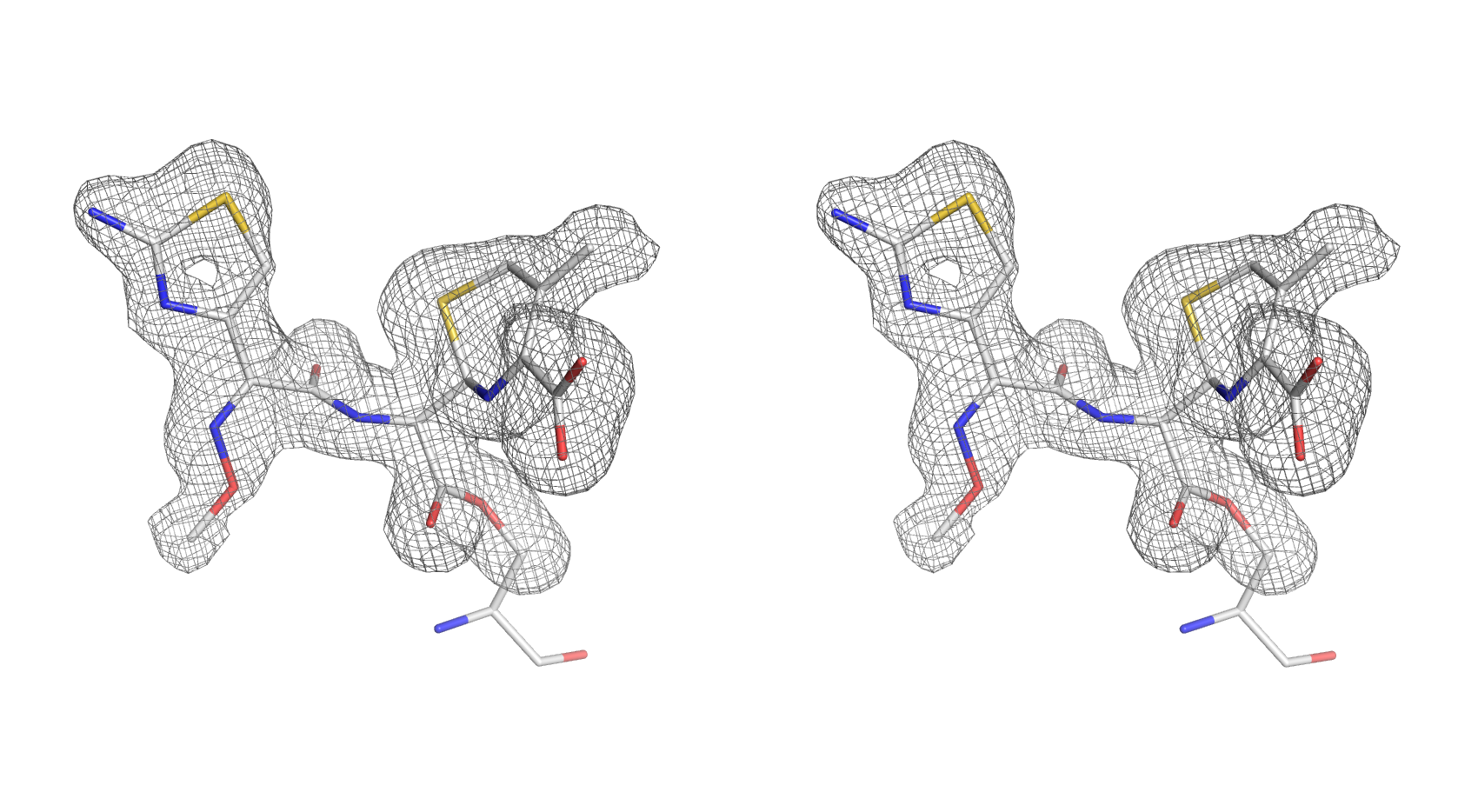

Supplement: Supplemental Table S1 [file mmc1.docx]
