# Supplementary material for: Conformational flexibility in carbapenem hydrolysis drives substrate specificity of the class D carbapenemase OXA-24/40
Source: J Biol Chem. 2022 Jun 14;298(7):102127. doi: 10.1016/j.jbc.2022.102127 (PMC9293634; doi:10.1016/j.jbc.2022.102127)
Supplement: Supplemental Figure S1A–H [file mmc2.docx]

**Supplementary Table 1.** Crystallographic statistics of OXA-24/40 complexes with ligands

|  | K84D imipenem | V130D imipenem | K84D meropenem | V130D meropenem | K84D ertapenem | V130D ertapenem | Wild-type ertapenem |
| --- | --- | --- | --- | --- | --- | --- | --- |
| Cell constants (Å;°) | *a*=*b*=102.16 | *a*=*b*=102.30 | *a*=*b*=102.77 | *a*=*b*=102.39 | *a*=*b*=102.83 | *a*=*b*=102.70 | *a*=*b*=102.81 |
|  | *c*=85.66 | *c*=86.92 | *c*=86.02 | *c*=85.65 | *c*=87.43 | *c*=85.80 | *c*=84.38 |
|  | *α*=*β*=*γ*=90 | *α*=*β*=*γ*=90 | *α*=*β*=*γ*=90 | *α*=*β*=*γ*=90 | *α*=*β*=*γ*=90 | *α*=*β*=*γ*=90 | *α*=*β*=*γ*=90 |
| Space group | P 4_1_2_1_2 | P 4_1_2_1_2 | P 4_1_2_1_2 | P 4_1_2_1_2 | P 4_1_2_1_2 | P 4_1_2_1_2 | P 4_1_2_1_2 |
| Resolution (Å) | 102.16–2.28 | 66.24–1.94 | 86.02–2.27 | 102.39–1.97 | 50.0–2.58 | 102.70–1.95 | 84.38–1.53 |
|  | (2.278–2.285) | (1.938–1.945) | (2.274–2.282) | (1.972–1.979) | (2.58-2.67) | (1.948–1.955) | (1.528–1.533) |
| Unique reflections | 20,836 (209) | 34,803 (331) | 21,722 (201) | 29,280 (307) | 14,713 (1,484) | 33,705 (339) | 68,587 (698) |
| Total reflections | 171,986 (1,752) | 249,722 (2,426) | 172,262 (1,574) | 211,465 (2,196) | 114,663 | 247,955 (2,494) | 555,353 (5,754) |
| R_merge_ (%) | 9.1 (61.7) | 10.3 (167.9) | 10.4 (110.9) | 14.0 (116.9) | 12.0 (64.8) | 8.8 (90.9) | 5.2 (87.7) |
| Rpim (%) (all I + I-) | 3.3(22.2) | 4.1 (65.5) | 3.9 (41.2) | 5.4 (46.1) | 4.7 (24.3) | 3.4 (35.9) | 1.9 (32.1) |
| CC(1/2) | 0.999 (0.672) | 0.998 (0.531) | 0.998 (0.896) | 0.997 (0.602) | 0.947 (0.985) | 0.997 (0.817) | 100 (86.8) |
| Completeness (%)^b^ | 97.5 (100.0) | 100.0 (98.5) | 99.8 (100.0) | 89.4 (92.5) | 96.1 (100.0) | 98.7 (98.5) | 99.6 (95.7) |
| <I/σ>^a^ | 18.0 (3.6) | 13.9 (1.3) | 16.3 (2.2) | 12.0 (2.2) | 15.8 (3.5) | 12.3 (2.3) | 21.9 (2.0) |
| Resolution for refinement (Å) | 102.16 – 2.28 | 66.24 – 1.94 | 86.02 – 2.27 | 102.39 – 1.97 | 46.03 – 2.58 | 72.62 – 1.95 | 65.31 – 1.53 |
| No. of protein residues | 244 | 244 | 245 | 244 | 244 | 244 | 245 |
| No. of waters | 69 | 95 | 53 | 68 | 35 | 100 | 257 |
| RMSD bond lengths (Å) | 0.008 | 0.011 | 0.008 | 0.011 | 0.007 | 0.010 | 0.016 |
| RMSD bond angles (°) | 1.67 | 1.92 | 1.74 | 1.92 | 1.95 | 1.80 | 2.09 |
| R-factor (%) | 18.5 | 19.7 | 21.1 | 19.9 | 21.5 | 18.8 | 17.2 |
| R_free_ (%)^c^ | 23.6 | 22.8 | 26.9 | 22.9 | 28.6 | 22.3 | 19.3 |
| Average B factor, protein (Å^2^) | 50.74 | 39.70 | 63.61 | 46.63 | 54.66 | 43.81 | 24.62 |
| Average B factor, ligand (Å^2^) | 58.00 | 47.54 | 79.62 | 64.36 | 68.59 | 50.64 | 37.84 |
| Average B factor, waters (Å^2^) | 50.14 | 43.86 | 61.50 | 47.63 | 51.82 | 47.8 | 36.94 |

|  | Wild-type doripenem | K84D cefotaxime |
| --- | --- | --- |
| Cell constants (Å;°) | *a*=*b*=102.70 | *a*=*b*=102.45 |
|  | *c*=84.47 | *c*=86.35 |
|  | *α*=*β*=*γ*=90 | *α*=*β*=*γ*=90 |
| Space group | P 4_1_2_1_2 | P 4_1_2_1_2 |
| Resolution (Å) | 50.00-1.90 | 50.00–1.62 |
|  | (1.97-1.90) | (1.68–1.62) |
| Unique reflections | 35,933 (3,404) | 58,160 (5,634) |
| Total reflections | 565,635 | 448,229 |
| R_merge_ (%) | 8.4 (32.0) | 9.7 (76.0) |
| Rpim (%) (all I + I-) | 2.0 (10.7) | 2.7 (45.3) |
| CC(1/2) | 0.994 (0.972) | 0.999 (0.838) |
| Completeness (%)^b^ | 99.5 (96.5) | 98.6 (97.2) |
| <I/σ>^a^ | 21.36 (6.53) | 11.58 (2.97) |
| Resolution for refinement (Å) | 23.63-1.90 | 27.76-1.62 |
| No. of protein residues | 245 | 244 |
| No. of waters | 190 | 305 |
| RMSD bond lengths (Å) | 0.013 | 0.013 |
| RMSD bond angles (°) | 1.97 | 2.01 |
| R-factor (%) | 17.2 | 17.8 |
| R_free_ (%)^c^ | 20.5 | 19.9 |
| Average B factor, protein, (Å^2^) | 31.98 | 27.67 |
| Average B factor, ligand (Å^2^) | 39.82 | 37.58 |
| Average B factor, waters (Å^2^) | 42.84 | 40.15 |
